# Supplementary figures and images for: Alcelaphine herpesvirus 1 genes A7 and A8 regulate viral spread and are essential for malignant catarrhal fever
Source: PLoS Pathog. 2020 Mar 16;16(3):e1008405. doi: 10.1371/journal.ppat.1008405 (PMC7098659; doi:10.1371/journal.ppat.1008405)

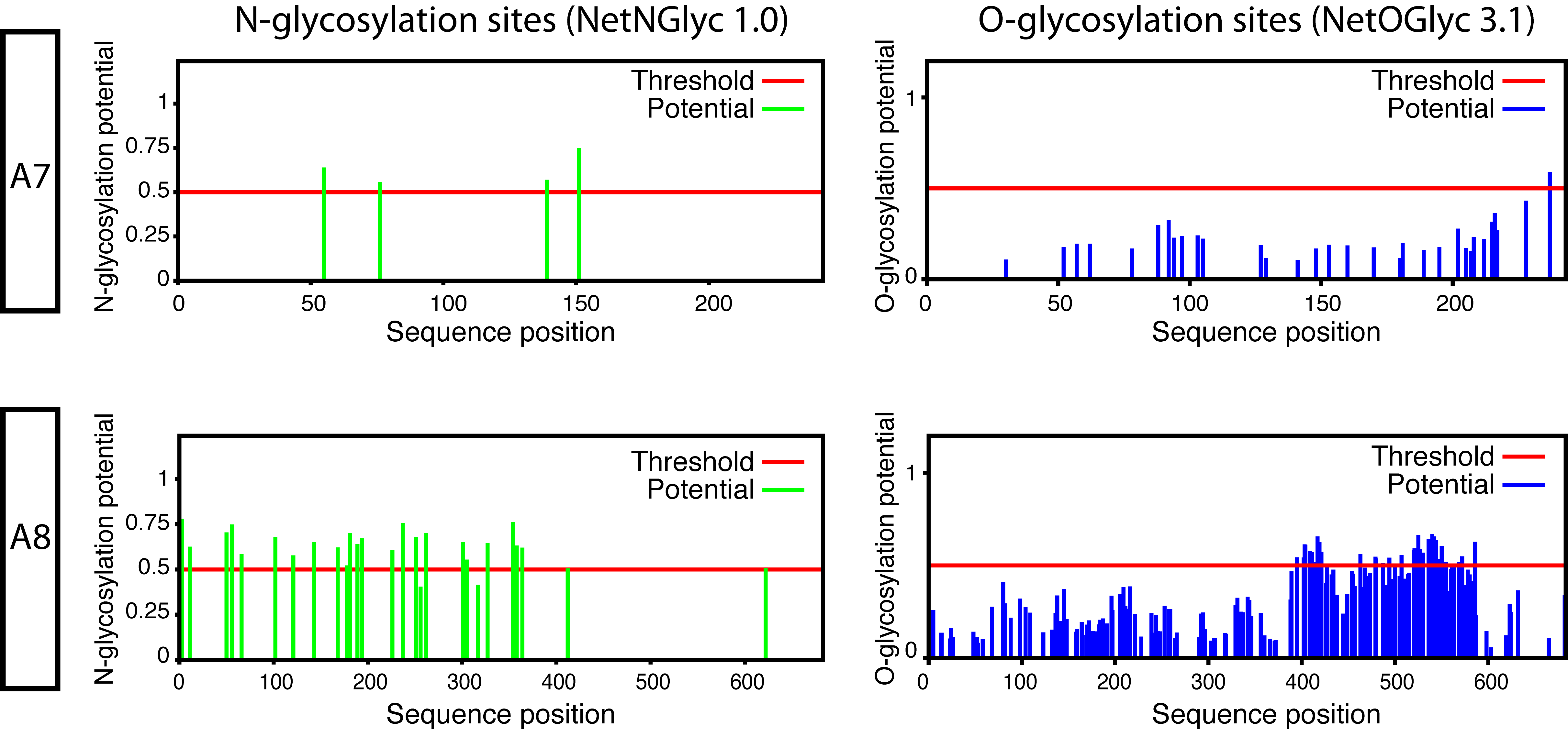

Supplement: S1 Fig — Glycosylation sites were predicted using NetNGlyc 1.0 (http://www.cbs.dtu.dk/services/NetNGlyc/) and NetOGlyc 4.0 (http://www.cbs.dtu.dk/services/NetOGlyc/) glycosylation site predictors. (TIF) [file ppat.1008405.s001.tif]

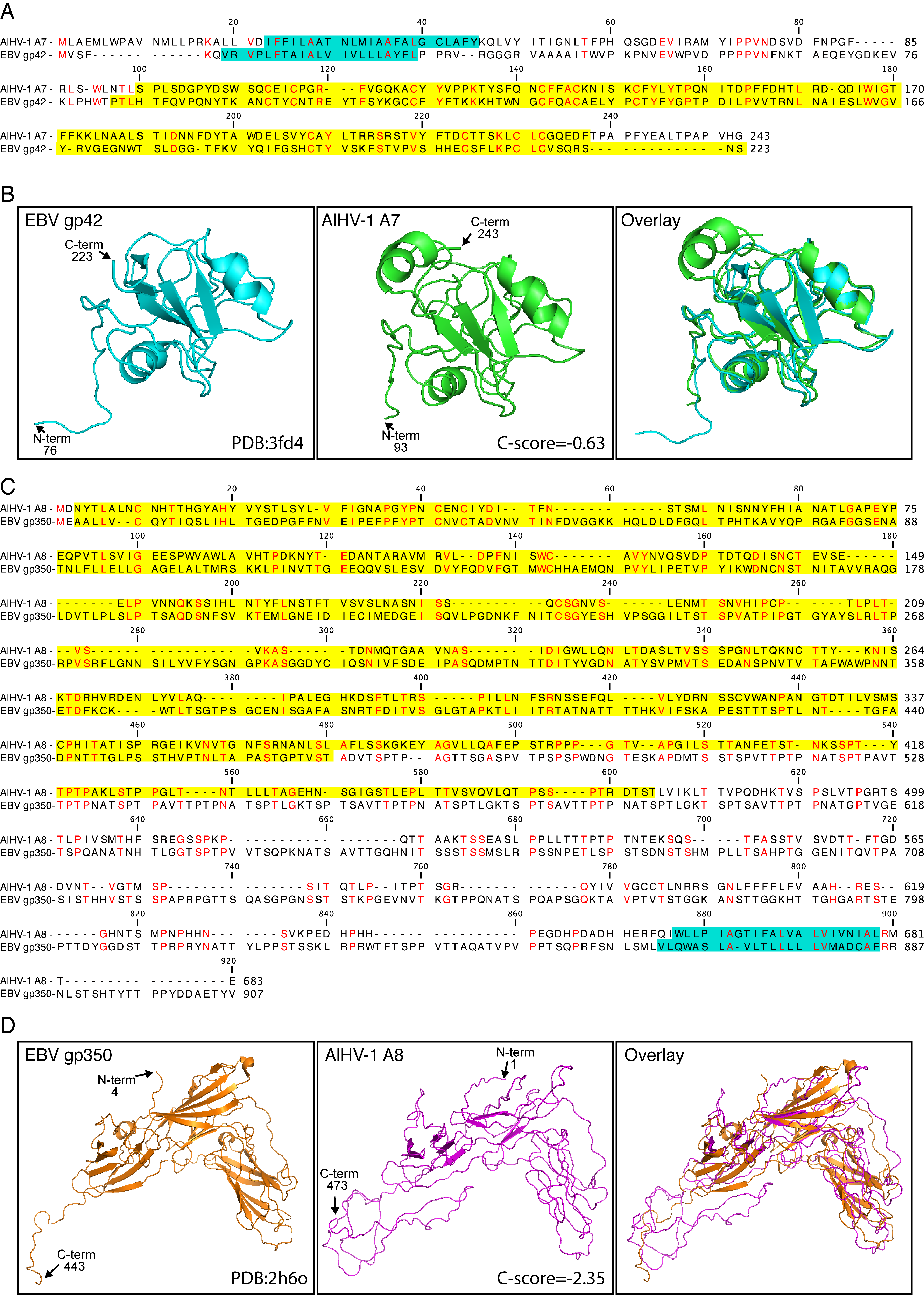

Supplement: S2 Fig — (A) Clustal Omega was used to align the primary amino acid sequences of the A7 protein and EBV gp42. The C-type lectin-like domain is highlighted in yellow (InterPro Protein sequence analysis & classification - https://www.ebi.ac.uk/interpro/). Conserved residues are shown in red. (B) Prediction of the three-dimensional structure of the extracellular domain of the A7 protein based on the structure of EBV gp42 [53]. (C) Clustal Omega was used to align the primary amino acid sequences of the A8 protein and EBV gp350. The extracellular domain is highlighted in yellow. (D) Prediction of the three-dimensional structure of the extracellular domain of the A8 protein based on the structure of EBV gp350 [47]. Transmembrane domains are highlighted in cyan (TMHMM Server v. 2.0), and conserved residues are shown in red. (TIF) [file ppat.1008405.s002.tif]

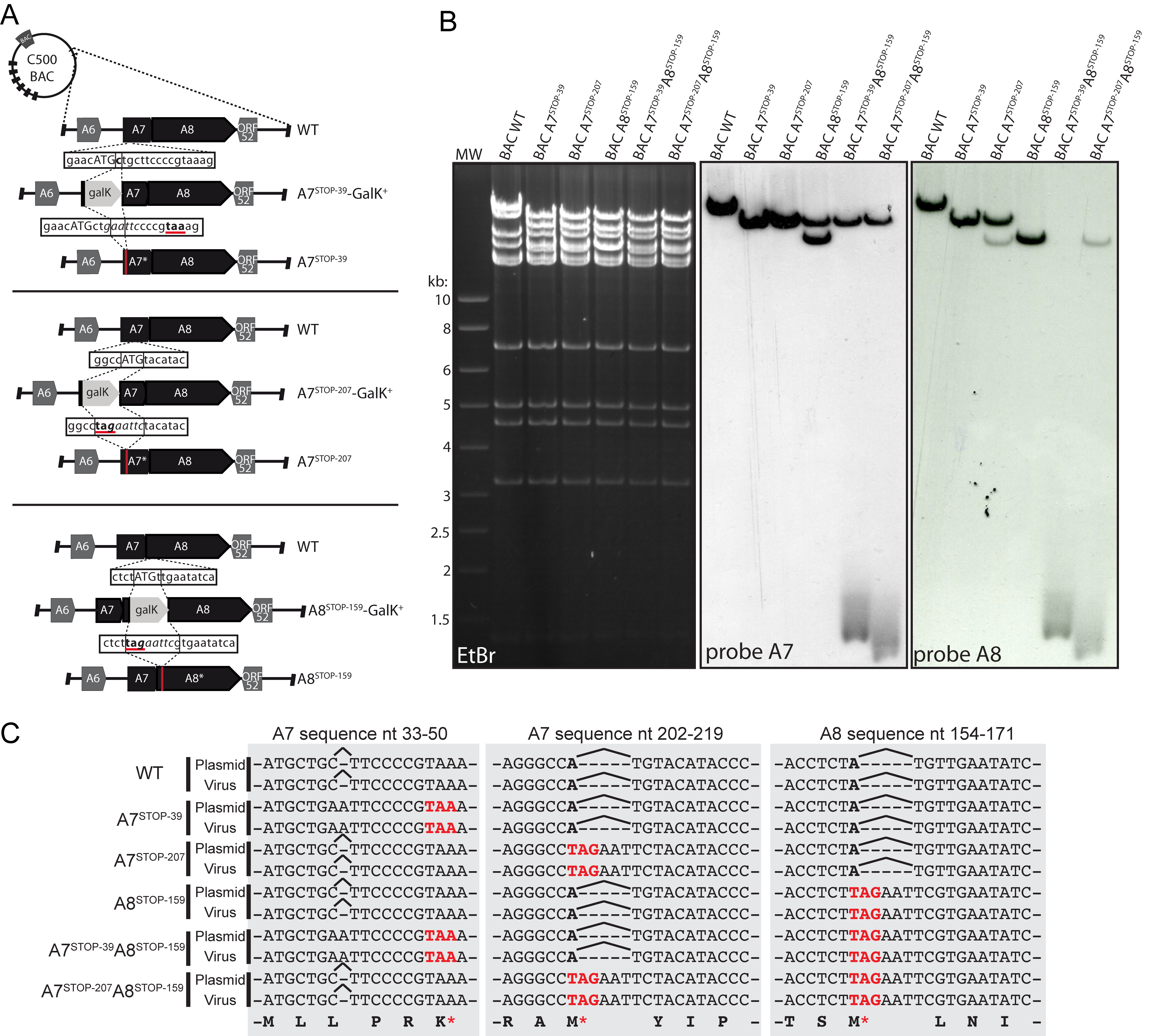

Supplement: S3 Fig — (A) The C500 BAC clone (WT) was used to produce the A7STOP-39, A7STOP-207, A8STOP-159, A7STOP-39A8STOP-159 and A7STOP-207A8STOP-159 BAC clones by mutagenesis. Galactokinase (galK)-based recombineering methodology for positive and negative selection was used to introduce an in-frame stop codon into the A7 or A8 coding sequence followed by an EcoRI restriction site (in italics). The stop codons generated are in bold font and underlined in red. A7STOP-39A8STOP-159 and A7STOP-207A8STOP-159 BAC clones were produced from A8STOP-159 BAC clone. (B) Southern blotting of the BAC clones generated. The A7 and A8 probes were produced by PCR as described in Methods. EtBr indicates ethidium bromide-stained lanes prior to blotting. The entire gel and Southern blots are displayed. (C) Sequence alignments of the mutagenesis performed to generate the mutants and the sequencing data obtained for BAC clones (Plasmid) and the BAC− viruses (Virus) generated from them. The first nucleotide of start codons is shown in bold black font and the stop codons are shown in bold red font. The positions of frameshifts in amino acid sequences are shown by bold red asterisks. (TIF) [file ppat.1008405.s003.tif]

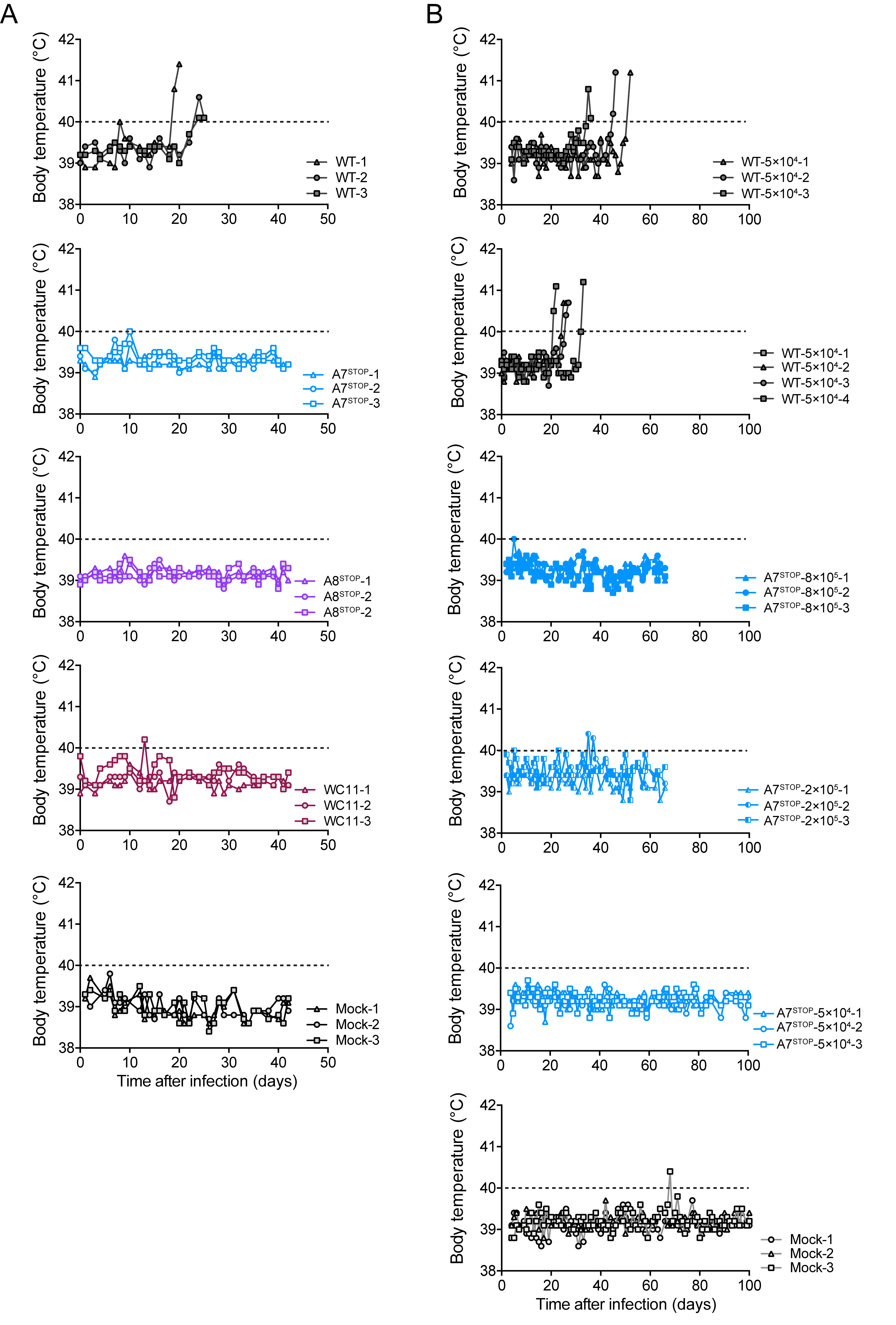

Supplement: S4 Fig — (A) Rabbits were infected by intravenous inoculation with 50 cm2 of mock-infected BT cells or cells infected with C500 BAC WT, A7STOP-207, A8STOP-159 or WC11 virus. (B) Rabbits were infected by intranasal inoculation of different doses (5×10^4^, 2×10^5^ or 8×10^5^ PFU per rabbit) of C500 BAC WT and A7STOP-207 virus. (TIF) [file ppat.1008405.s004.tif]

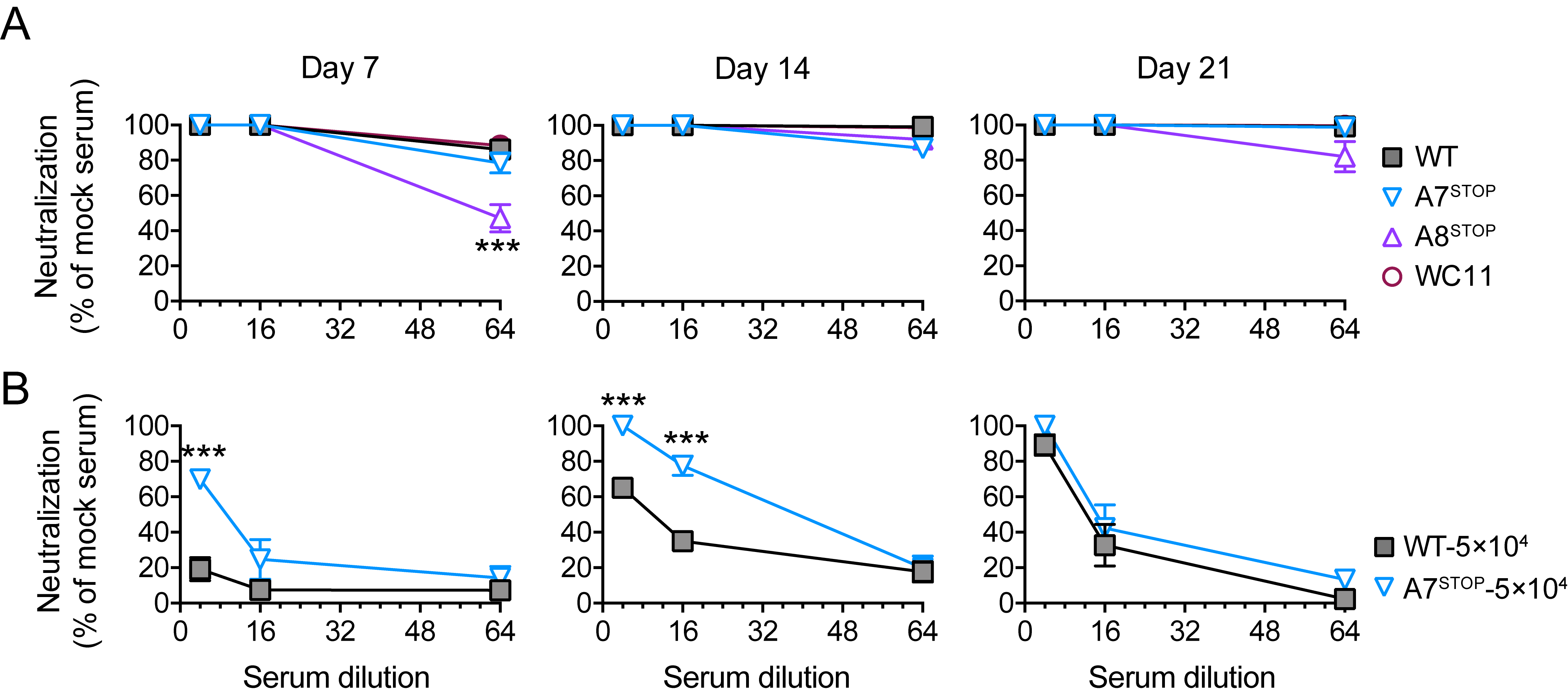

Supplement: S5 Fig — (A) Rabbits were infected by intravenous inoculation with 50 cm2 of mock-infected BT cells or cells infected with C500 BAC WT, A7STOP-207, A8STOP-159 or WC11 virus. (B) Rabbits were infected by intranasal inoculation of 5×10^4^ PFU per rabbit of C500 BAC WT and A7STOP-207virus. Serum samples were tested for neutralizing antibodies at day 7, 14 and 21. (TIF) [file ppat.1008405.s005.tif]

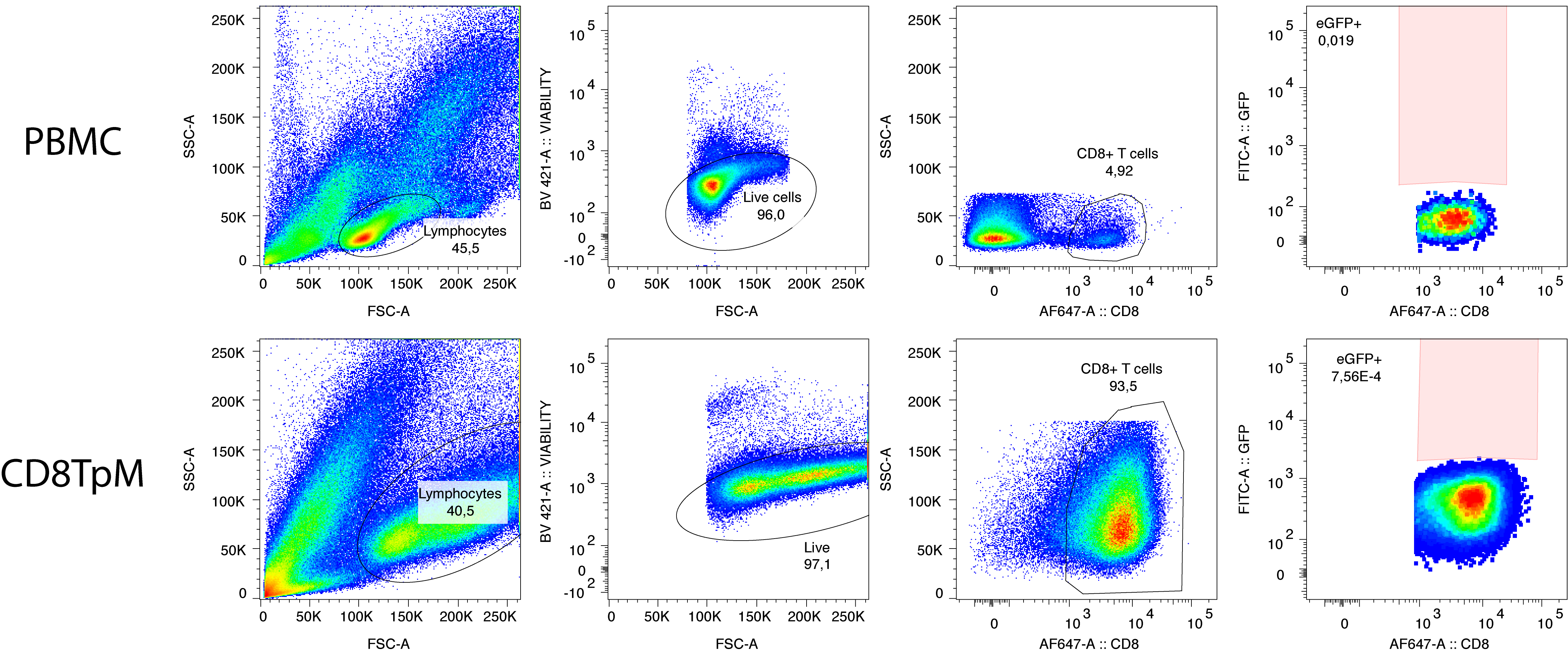

Supplement: S6 Fig — (TIF) [file ppat.1008405.s006.tif]
