# Supplementary material for: Alcelaphine herpesvirus 1 genes A7 and A8 regulate viral spread and are essential for malignant catarrhal fever
Source: PLoS Pathog. 2020 Mar 16;16(3):e1008405. doi: 10.1371/journal.ppat.1008405 (PMC7098659; doi:10.1371/journal.ppat.1008405)
Supplement: S1 Table — (PDF) [file ppat.1008405.s007.pdf]

Table S1. AlHV-1 strain WC11 genome organization compared to other macaviruses

WC11	C500	AlHV-2	OvHV-2	BoHV-6	% ID of AlHV1 proteins	AlHV1 protein name	Coding sequence comment	Description
	A1					protein A1		
				Bov1.b1				
				Bov1.b2				
				Bov1.b3				
A2	A2	A2	Ov2	Bov2	99	protein A2		bZIP domain
		A2.5	Ov2.5					
				Bov2.b1				
				Bov2.b2				
				Bov2.b3				
A3	A3	A3	Ov3		100	semaphorin A3	Semaphorin family	signal peptide; possibly involved in intracellular signalling
A4	A4	A4	Ov3.5		100	protein A4		signal peptide
3	3	3	3	3	99.6	protein G3	FGARAT family	
A4.5	A4.5	A4.5	Ov4.5	Bov4.5	100	apoptosis regulator BALF1	Bcl-2 family	involved in apoptosis
6	6	6	6	6	99.9	single-stranded DNA-binding protein		Zn-finger; involved in DNA replication; possibly involved in gene regulation
7	7	7	7	7	99.7	DNA packaging terminase subunit 2		involved in DNA encapsidation
8	8	8	8	8	99.9	envelope glycoprotein B		type 1 membrane protein; possible membrane fusogen; binds cell surface heparan sulphate; involved in cell entry; involved in cell-to-cell spread
9	9	9	9	9	99.8	DNA polymerase catalytic subunit		involved in DNA replication
A5	A5	A5	Ov5	Bov5	99.3	membrane protein BILF1	GPCR family	type 3 membrane protein; involved in intracellular signalling
10	10	10	10	10	99.5	protein G10	DURP family	
11	11	11	11	11	99.8	virion protein G11	DURP family	
17.5	17.5	17.5	17.5	17.5	99.3	capsid scaffold protein		clipped near C terminus; involved in capsid morphogenesis
17	17	17	17	17	99.2	capsid maturation protease		serine protease (N-terminal region); minor scaffold protein (remainder of protein, clipped near C terminus); involved in capsid morphogenesis
18	18	18	18	18	99.3	protein UL79		promotes accumulation of late transcripts
19	19	19	19	19	99.6	DNA packaging tegument protein UL25		located on capsid near vertices; possibly stabilizes the capsid and retains the genome; involved in DNA encapsidation
20	20	20	20	20	100	nuclear protein UL24		
21	21	21	21	21	100	thymidine kinase		involved in nucleotide metabolism
22	22	22	22	22	99.7	envelope glycoprotein H		type 1 membrane protein; possible membrane fusogen; complexed with envelope glycoprotein L; involved in cell entry; involved in cell-to-cell spread
23	23	23	23	23	99	tegument protein UL88		
24	24	24	24	24	99.7	protein UL87		promotes accumulation of late transcripts
25	25	25	25	25	99.9	major capsid protein		6 copies form hexons, 5 copies form pentons; involved in capsid morphogenesis
26	26	26	26	26	100	capsid triplex subunit 2		complexed 2:1 with capsid triplex subunit 1 to connect capsid hexons and pentons; involved in capsid morphogenesis
27	27	27	27	27	99.3	envelope glycoprotein 48		type 2 membrane protein involved in cell-to-cell spread
				28				
29	29	29	29	29	99.9	DNA packaging terminase subunit 1		contains ATPase domain; involved in DNA encapsidation
30	30	30	30	30	100	protein UL91		
31	31	31	31	31	100	protein UL92		
32	32	32	32	32	100	DNA packaging tegument protein UL17		capsid-associated; involved in DNA encapsidation; involved in capsid transport
33	33	33	33	33	100	tegument protein UL16		possibly involved in virion morphogenesis
34	34	34	34	34	99.7	protein UL95		promotes accumulation of late transcripts
35	35	35	35	35	100	tegument protein UL14		involved in virion morphogenesis
36	36	36	36	36	99.8	tegument serine/threonine protein kinase		involved in protein phosphorylation
37	37	37	37	37	100	deoxyribonuclease		involved in DNA processing
38	38	38	38	38	100	myristylated tegument protein		envelope-associated; involved in virion morphogenesis
39	39	39	39	39	100	envelope glycoprotein M		type 3 membrane protein; 8 transmembrane domains; complexed with envelope glycoprotein N; involved in virion morphogenesis; involved in membrane fusion
40	40	40	40	40	99.5	helicase-primase subunit		involved in DNA replication
42	42	42	42	42	100	tegument protein UL7		involved in virion morphogenesis
43	43	43	43	43	99.8	capsid portal protein		dodecamer located at one capsid vertex in place of penton; involved in DNA encapsidation

44	44	44	44	44	100	**helicase-primase helicase subunit**		involved in DNA replication
45	45	45	45	45	100	**tegument protein G45**		
46	46	46	46	46	100	**uracil-DNA glycosylase**		involved in DNA repair
47	47	47	47	47	100	**envelope glycoprotein L**		contains signal peptide; complexed with envelope glycoprotein H; involved in cell entry; involved in cell-to-cell spread
48	48	48	48	48	99.6	**tegument protein G48**		
50	50	50	50	50	99.7	**protein Rta**		involved in gene regulation
			49	49				
**A6**	**A6**	**A6**	**Ov6**	**Bov6**		**protein Zta**		ZEBRA, Zta, Z; bZIP protein; involved in gene regulation
	**A7**	**A7**	**Ov7**	**Bov7**		**envelope glycoprotein 42**		complexed with envelope glycoprotein H and envelope glycoprotein L; involved in cell entry
	**A8**	**A8**	**Ov8**	**Bov8**		**envelope glycoprotein 350**		type 1 membrane protein; involved in cell attachment
52	52	52	52	52	100	**virion protein G52**		
53	53	53	53	53	100	**envelope glycoprotein N**		type 1 membrane protein; complexed with envelope glycoprotein M; involved in virion morphogenesis; involved in membrane fusion
54	54	54	54	54	100	**deoxyuridine triphosphatase**	DURP family	involved in nucleotide metabolism
55	55	55	55	55	100	**tegument protein UL51**		involved in virion morphogenesis
56	56	56	56	56	100	**helicase-primase primase subunit**		involved in DNA replication
57	57	57	57	57	100	**multifunctional expression regulator**		RNA-binding protein; shuttles between nucleus and cytoplasm; inhibits pre-mRNA splicing; exports virus mRNA from nucleus; exerts most effects post-transcriptionally; involved in gene regulation; involved in RNA metabolism and transport
58	58	58	58	58	99.7	**envelope protein UL43**		type 3 membrane protein; possibly involved in membrane fusion
59	59	59	59	59	100	**DNA polymerase processivity subunit**		dsDNA-binding protein; involved in DNA replication
60	60	60	60	60	100	**ribonucleotide reductase subunit 2**		involved in nucleotide metabolism
61	61	61	61	61	100	**ribonucleotide reductase subunit 1**		involved in nucleotide metabolism
62	62	62	62	62	100	**capsid triplex subunit 1**		complexed 1:2 with capsid triplex subunit 2 to connect capsid hexons and pentons; involved in capsid morphogenesis
63	63	63	63	63	99.7	**tegument protein UL37**		complexed with large tegument protein; involved in virion morphogenesis
64	64	64	64	64	100	**large tegument protein**		complexed with tegument protein UL37; ubiquitin-specific protease (N-terminal region); involved in capsid transport
65	65	65	65	65	100	**small capsid protein**		located externally on capsid hexons; involved in capsid morphogenesis; possibly involved in capsid transport
66	66	66	66	66	99.8	**protein UL49**		
67	67	67	67	67	100	**nuclear egress membrane protein**		type 2 membrane protein; interacts with nuclear egress lamina protein; involved in nuclear egress
67A	67A	67A	67A	67A	100	**DNA packaging protein UL33**		interacts with DNA packaging terminase subunit 2; involved in DNA encapsidation
68	—68	68	68	68	100	**DNA packaging protein UL32**		involved in DNA encapsidation; possibly involved in capsid transport
69	69	69	69	69	100	**nuclear egress lamina protein**		interacts with nuclear egress membrane protein; involved in nuclear egress
73	73	73	73	73	91.3	**nuclear antigen LANA-1**		chromosome-tethering protein; involved in latency
75	75	75	75	75	99.5	**tegument protein G75**	FGARAT family	
**A9**	**A9**	**A9**	**Ov9**	**Bov9**	100	**apoptosis regulator A9**	Bcl-2 family	involved in apoptosis
**A9.5**	**A9.5**	**A9.5**	**Ov9.5**		82.4	**glycoprotein A9.5**		secreted protein
**A10**	**A10**	**A10**	**Ov10**		92.8	**membrane protein A10**		
				**Bov9.b1**				
				**Bov9.b2**				
