# Supplementary material for: Alcelaphine herpesvirus 1 genes A7 and A8 regulate viral spread and are essential for malignant catarrhal fever
Source: PLoS Pathog. 2020 Mar 16;16(3):e1008405. doi: 10.1371/journal.ppat.1008405 (PMC7098659; doi:10.1371/journal.ppat.1008405)
Supplement: S2 Table — (PDF) [file ppat.1008405.s008.pdf]

Table S2. Whole AlHV-1 genome sequencing data

Description	Total reads (no.)	Target reads (no.)	Target reads (%)	Coverage (reads/nt)
A7$^{STOP-39}$ (BAC plasmid)	1,352,076	1,333,326	99	1,366
A7$^{STOP-207}$ (BAC plasmid)	1,336,896	1,322,936	99	1,355
A8$^{STOP-159}$ (BAC plasmid)	1,516,540	1,482,871	98	1,519
A7$^{STOP-207}$ (BAC^{-} virus in BT cells)	1,460,388	251,143	17	275
A8$^{STOP-159}$ (BAC^{-} virus in BT cells)	1,520,344	165,427	11	181
