# Supplementary material for: Alcelaphine herpesvirus 1 genes A7 and A8 regulate viral spread and are essential for malignant catarrhal fever
Source: PLoS Pathog. 2020 Mar 16;16(3):e1008405. doi: 10.1371/journal.ppat.1008405 (PMC7098659; doi:10.1371/journal.ppat.1008405)
Supplement: S3 Table — (PDF) [file ppat.1008405.s009.pdf]

Table S3. Oligonucleotides.

Sequence	Primer	Primer sequence (5' – 3')
galK sequence	A7-NS-galKFwd39	CTGGGCTCTGAGATGTTGGCAGAAATGTTGTGGCCTGC AGTGAACATGCTGCCTGTTGACAATTAATCATCGGCA
	A7-NS-galKRev39	GTGGCTGCCAAAATAAAAAAAATGTCTACCAAAAGTGCT TTACGGGGAATCAGCACTGTCCTGCTCCTT
galK sequence	A7-NS-galkFwd207	GGCAACCTAACCTTTCCCCACCAGAGCGGGGATGAGG TTATTAGGGCCCCTGTTGACAATTAATCATCGGCA
	A7-NS-galkRev207	GTCTGAACCCAGGGTTAAAATCCACACTGTCATTGACT GGGGGTATGTATCAGCACTGTCCTGCTCCTT
galK sequence	A8-NS-galkFwd	CCAAACTGTGAAAACTGTATCTATGACATAACCTTCAAC AGCACCTCTCCTGTTGACAATTAATCATCGGCA
	A8-NS-galkRev	GGCTCCGAGGGTGGCATTAGCTATATGGAAATAATTGT TGCTGATATTCATCAGCACTGTCCTGCTCCTT
A7	A7-NS-oligoFwd39	CTGGGCTCTGAGATGTTGGCAGAAATGTTGTGGCCTGC AGTGAACATGCTGAATTCCCCGTAAAGCACTTTTGGTAG ACATTTTTTTTATTTTGGCAGCCAC
	A7-NS-oligoRev39	GTGGCTGCCAAAATAAAAAAAATGTCTACCAAAAGTGCT TTACGGGGAATTCAGCATGTTCACTGCAGGCCACAACA TTTCTGCCAACATCTCAGAGCCCAG
A7	A7-NS-oligoFwd207	GGCAACCTAACCTTTCCCCACCAGAGCGGGGATGAGG TTATTAGGGCCTAGAATTCTACATACCCCCAGTCAATGA CAGTGTGGATTTTAACCCTGGGTTCAGAC
	A7-NS-oligoRev207	GTCTGAACCCAGGGTTAAAATCCACACTGTCATTGACT GGGGGTATGTAGAATTCTAGGCCCTAATAACCTCATCC CCGCTCTGGTGGGGAAAGGTTAGGTTGCC
A8	A8-NS-oligoFwd	CCAAACTGTGAAAACTGTATCTATGACATAACCTTCAAC AGCACCTCTTAG*AATTCG*TGAATATCAGCAACAATTATT TCCATATAGCTAATGCCACCCTCGGAGCC
	A8-NS-oligoRev	GGCTCCGAGGGTGGCATTAGCTATATGGAAATAATTGT TGCTGATATTCACGAATTCTAAGAGGTGCTGTTGAAGGT TATGTCATAGATACAGTTTTCACAGTTTGG
A7	A7-165F	CTAACCTTTCCCCACCAGAGC
	A7-303R	CTCGCACTGGGACCAAGAGTC
	A7-476F	CACACCTTACGAGACCAGG
	A7-deltastopR	TCCATGGACGGGTGCTG
A8	A8-ATGF	ATGGATAACTATACACTAGC
	A8-278R	CAGGCGTATGTACTGCGAGC
	A8-1661F	CACTGTATCAGTGGATACTAC
	A8-deltastopR	TTCTGTCATACGTAGAGCTA
