# Supplementary material for: Alcelaphine herpesvirus 1 genes A7 and A8 regulate viral spread and are essential for malignant catarrhal fever
Source: PLoS Pathog. 2020 Mar 16;16(3):e1008405. doi: 10.1371/journal.ppat.1008405 (PMC7098659; doi:10.1371/journal.ppat.1008405)
Supplement: Attachment — Submitted filename: Response to reviewers.pdf [file ppat.1008405.s010.pdf]

Dr Benjamin Dewals, DVM, PhD
Group leader - Research Associate of the F.R.S-FNRS
Faculty of Veterinary Medicine
Immunology-Vaccinology
Tel. +32 4 366 42 69
Fax. +32 4 366 42 61
bgdewals@uliege.be

Response to reviewer's comments : Ms # PPATHOGENS-D-19-01549

Reviewer #1:
This paper describes an analysis of the attenuated AlHV1 strain WC11 and of the production and phenotypes of two BAC clone with stop mutations in genes deleted in WC11. The study is an important step forward in understanding the basis of attenuation in MCF viruses. It provides a well written and thorough analysis of the A7 and A8 genes, showing that these proteins mediate cell-free and cell-associated modes of propagation and that loss of either function leads to loss of virulence for MCF.

The paper is suitable for publication in its current state but may benefit from minor modifications as described in the comments for authors.

Author's response : we are thankful to this reviewer who appreciated the relevance of our study. We provide below information on how we have addressed the requests for minor revisions.

Minor comments :
Line 150. Please be explicit. Clarify whether the LUR or TR copies are meant.
Response: We have now clarified that both LUR and TR are meant (line 156, revised manuscript).

Line 153. Please specify German WC11 stock to distinguish from the stock sequenced here
Response: we have now specified that we are referring to the German WC11 stock (line 159, revised manuscript).

Line 157-8. please clarify text here, as this point may be unclear to some readers
Response: We have added the following sentence of the revised manuscript to make the point clear to the reader. "*The duplicated sequence present in the TR also contained the same deleted sequence, indicating that the deletion had occurred prior to the duplication event [36]***." (lines 163-165, revised manuscript)**

Line 160. "during multiple passages" rather than 'following multiple passages'
Response: We have made the change (line 167, revised manuscript).

Lines 194-209. the authors should acknowledge previous publications describing the similarity between A7/A8 and gp42/350 (Russell, 2014. In: Manual of Security Sensitive Microbes and Toxins. CRC Press; https://www.crcpress.com/Manual-of-Security-Sensitive-Microbes-and-Toxins/Liu/p/book/9780367378745); and the function of the A8 homolog Ov8 in membrane fusion (AlHajri, 2017. J.Virol. 91:e02454-16. https://doi.org/10.1128/JVI.02454-16).
Response: We thank the reviewer for the suggestion and we have added both references in the text (lines 203 ref 41 – and 210-211, the revised manuscript).

Line 217. In the BAC mutation description it is important that you show that both TR and LUR gene copies of A7 & A8 were mutated, or explain why this was not required
Response: We thank the reviewer for this suggestion. However, although the first 138 bp of A7 coding sequence is present in the duplicated region in the TR, A8 is not duplicated (see ref. 36: Myster et al., 2016 Scientific Reports). We have added the necessary information in lines 237-241 of the revised manuscript that the insertion of the stop codon in position "39" of A7 was only found in the LUR, not the duplicated part

of A7 in the TR. This observation led us to use the A7$^{STOP-207}$ mutant in order to avoid the risk of reversion from the duplicated region. We have also identified in Fig. 1 the 2 duplicated regions of C500 and WC11.

Line 251. Fluorescence microscopy and use of antibody 15A are not mentioned in methods
Response: We have clarified these methods in the revised manuscript (lines 552-556, revised manuscript).

Line 300. is ref 43 intended? Maybe should be 35?
Response: This is a mistake. We have changed it accordingly (line 353, revised manuscript)

Line 302. While it's completely acceptable to choose to study A7/A8, I think it is important that the authors mention why there was no focus on A1 in this work.
Response: We have added in the discussion (page 13, lines 353-359) an explanation justifying our choice to focus on A7 and A8.

Line 342. I don't feel that sup fig 4 is necessary
Response: We have removed Supplementary Figure S4.

Reviewer #2:
Myster and coauthors address the genetic differences between the pathogenic C500 and an attenuated WC11 strain of the Macavirus Alcelaphine herpesvirus 1 (AlHV-1). AlHV-1 and Ovine Herpesvirus 2 induces malignant catarrhal fever in susceptible ruminants and in rabbits. In cattle and rabbits, MCF is characterized by proliferation of CD8+ T-cells, a lymphoproliferative disease with strong similarity to the pathology caused by the New World primate viruses herpesvirus saimiri and ateles in tamarins and marmosets (which is overlooked in the introduction). They focus on one of three major differences between the pathogenic and the attenuated strains, the putative glycoproteins encoded by ORFs A7 and A8, which are both lacking in the attenuated WC11 strain. Individual bacmid-technology based mutants of each of A7 and A8 were generated in the C500 strain and compared to C500 and the A7 and A8 deleted WC11 in vitro and in vivo. Although no marker rescue viruses were made, the recombinants were sequenced and the data obtained with the mutants seems consistent. However, the important rabbit experiments do not discriminate between an inability to spread efficiently in the rabbits, failure to gain access to CD8 T cell populations or an inability to reprogram such cells toward lymphoproliferation.

The authors provide an extensive comparison of the replicative behavior of C500, WC11 and two A7, one A8 mutants and mutants. Herein, depending on the cell culture system, the A7 mutants show an intermediate phenotype, increasing cell free propagation, while A8 seems to be required for cell associated spread. This may e.g. reflect alternative receptor usage for entry in these culture systems.

Finally, rabbit experiments show that the C500 A7stop, A8stop and, as expected, attenuated WC11 do not induce MCF or CD8 T cell lymphoproliferation in this experimental model. They provide evidence all strains were able to infect rabbits, by serology and detection of viral genomes. The mutants and attenuated WC11, however, were only detectable at very low levels in the spleen, and not in circulating PBMC or lymph node.

Response:

We thank the reviewer for the thorough reading of our manuscript and the relevant comments.

First, we have added a reference to New World monkey herpesvirus Saimiri and Ateles (revised manuscript ref 20, lines 102-105).

Second, we agree with the major comment on tte lack of mechanistic insight was missing in the original manuscript which could explain why the absence of A7 or A8 renders AlHV-1 non-pathogenic (i.e. unable to induce MCF in the rabbit model). We agree with the three main hypotheses provided by the reviewer that the lack of A7 or A8 might:

(i) impair viral spread in vivo upon infection (and thus not reach target CD8$^+$ T cells)

(ii) impair viral entry into CD8$^+$ T cells

(iii) impair AlHV-1-induced proliferation of CD8$^+$ T cells

We now provide in the revised version of the manuscript additional data requested by the reviewer as well as unpublished data which support the hypothesis that absence of A7 or A8 affects viral spread and access to CD8 T cells (points i and ii), while being dispensable for actual reprogramming CD8 T cells (see response to major comments below).

Regarding point (iii): we have published microarray data in 2013 showing that A7 and A8 RNA could not be detected from the lymph nodes of MCF-developing calves (Palmeira et al., PNAS, 2013). This important information was recently supported by yet unpublished data. Indeed, we have performed an RNA sequencing analysis on CD8$^+$ T cells highly purified from peripheral blood of calves developing MCF and showing expansion of CD8$^+$ T cells. From these data, we could draw a coverage map of viral RNAs expressed in CD8$^+$ T cells throughout the genome of AlHV-1. While we could clearly detect the expression of a number of gene regions including latency-associated ORF73, we could not detect any sequencing read that would map the A7 or A8 coding sequence. This is an important information which strongly suggests that both genes are dispensable for CD8$^+$ T cell reprogramming during MCF (as they are not expressed in infected CD8$^+$ T cells during MCF). We are happy to share the actual data with the reviewer but would not include these confidential data in this response letter.

Major comments:

1. Why did they not make / analyze a double A7 and A8 stop mutant, corresponding to the situation in WC11? If the double mutant would show a replicative behavior even more similar to WC11, this would be important with respect to possible influence of the other two major genetic differences, which are located on the left and right genome ends, i.e. the absent putative ORF A1 and the variable ORFs A9.5 and A10?

Response:

We now provide growth data of a double A7^{STOP}A8^{STOP} mutant. Although the mutant was generated at the first submission, we did not include it in the initial experiments as we believed it might confuse the main message. However, we understand that if we want to address the potential role of the genomic rearrangements and sequence divergence of strain WC11, a double impairment of A7 and A8 is of interest.

Thus, we now have included the characterization of double A7^{STOP}A8^{STOP} mutant (revised Suppl. Figure S3). We have repeated the growth kinetics in BT and EBL cells (revised Fig. 3B), plaque size assay (revised Fig. 3C), viral propagation (revised Fig. 3D) and mAb 15-A (gp115 complex) immunostaining and plaque morphology (revised Fig. 4) using the A7$^{STOP-207}$A8$^{STOP-159}$ virus. Regarding the plaque size assay in BT cells, we

realized there was a mistake in the scale of the y-axis of Figure 3C, which is now adjusted and correct in the revised Figure 3.
The results show that the absence of both A7 and A8 expression does not render AlHV-1 even more similar to strain WC11 in term of growth in vitro in the selected cell lines. On the contrary double A7^{STOP}A8^{STOP} mutant displayed an intermediary level of fitness which was much comparable to strain C500 BAC WT in BT cells, whereas several characteristics shared with the single A7^{STOP} virus essentially in EBL cells. Indeed, we could observe significantly increased cell-free virions (revised Fig. 3B, bottom), smaller plaques in EBL cells (revised Fig. 3C, bottom) and the absence of syncytia formation (revised Fig. 4 in both BT and EBL cells). The manuscript has been revised accordingly on pages 9 and 10.

Thus, there must be additional mechanism(s) than absence of A7 and A8 expression to explain why strain WC11 growth is enhanced. The obvious candidates are A1 and microRNAs identified in this region (see ref. 21 Sorel et al, 2015) and the region encompassing A9.5 to part of A10 (no data yet available on viral growth). In addition, WC11 genome has retained some part of A8 sequence which corresponds to a potential 179 aa expressed transmembrane protein. We could not detect strong mRNA expression of this sequence in cells infected with WC11 (revised Fig. 2F) but it might still be a potential explanation due to the severe genomic rearrangement found in the region of ORF50/A6/A7/A8 that might have modified viral gene regulation. Investigating this further is however out of the scope of this manuscript (lines 390-398, revised manuscript).

Finally, as both A7 and A8 expression are independently essential to induce MCF (revised Figures 6-9), we did not include in vivo experiments using the double A7^{STOP}A8^{STOP} mutant.

2. The rabbit experiments do not discriminate between an inability to spread efficiently in the rabbits, failure to gain access to CD8 T cell populations or an inability to reprogram such cells toward lymphoproliferation. There was no monitoring for viral spread (genome copies in blood?) during the infection experiment, only at endpoint. Thereby, vastly different time points are compared, and the low levels of virus genomes observed with A7 and A8 stop mutants and WC11 may reflect long term immune control of latent infection, compared to an acute disease with multiple virus genomes in the CD8 population.
<u>Response:</u> We agree with the reviewer comment. While we had harvested PBMCs over time, we did not perform qPCR analysis to detect genome copies. The main reason was that in a rabbit (or a cow) infected with the pathogenic C500 strain, there is a long silent phase where AlHV-1 is not detectable in peripheral blood. Thus, investigating the ability of AlHV-1 to spread in rabbits is not straightforward.
Nonetheless, we have extracted DNA from cryopreserved (-80°C) PBMCs from each animal over the course of the infection (in both in vivo experiments) and performed quantitative PCR to detect AlHV-1 ORF3 genomic sequence. The results are shown in revised Figures 8C and 9C and demonstrate that viral genomes are undetectable at each selected time points in animals infected with strains WC11, A7^{STOP} or A8^{STOP}. In animals infected with strain C500 WT, viral genomes could be detected from about 2 to 3 weeks post-infection, which corresponds to a time-point when persistently infected CD8^{+} T cells start to proliferate.
In addition, we have extracted DNA from cryopreserved (-80°C) tissue biopsies of spleen, lung and liver and performed quantitative PCR to detect AlHV-1 ORF3 genomic sequence. Here, we could detect viral genomes in rabbits infected with strains WC11,

A7^{STOP} or A8^{STOP} at a significantly lower level compared to rabbits developing MCF after infection with the strain C500 WT.
We have revised the results and discussion sections in order to include these important additional results suggesting that AlHV-1 infection is present and persistent in probably some niches in absence of A7 or A8 while the access to CD8^{+} T cells is most probably inefficient (lines 303-310 and 419-427, revised manuscript).

3. In vitro infection (or by cocultivation) of bovine or rabbit CD8 T-cells with the EGFP marker viruses (or by infection followed by PCR) could demonstrate whether this is a matter of entry/access to this population.
Response: We thank the reviewer for the suggestion. We now provide data of a co-culture assay where BT or EBL cells were infected with the BAC+ (eGFP+) WT, A7^{STOP}, A7^{STOP} or A7^{STOP}A8^{STOP} viruses before co-culture of bovine PBMCs or *T. parva* immortalized bovine CD8^{+} T cells. The results clearly suggest that the absence of A7 and/or A8 does not impair infection of bovine CD8^{+} T cells. We have revised the manuscript accordingly: Figure 10 and result section pages 11 and 12 – lines 326-348 and 434-444)

4. Cells injected intravenously, if not phagocytosed or killed the immune cells, may end in small vessels of lung, spleen, liver etc. where they may infect neighboring tissue, not necessarily T cells. Similarly, after intranasal infection, local infection and spread is critical, and T cells are unlikely to be the first cells encountering a pathogen.
Response: This is a fair comment and we fully agree with the reviewer. The fact that the lack of A7 or A8 would impact viral spread is the focus of our study. Our in vitro experiments using nasal fibroblasts or embryonic lung cell lines provide evidence that propagation of AlHV-1 is affected due to absence of A7 or A8; and is a major hypothesis explaining why MCF is not induced in animals infected with A7^{STOP} or A8^{STOP} virions. This fourth major comment raised by the reviewer is in direct line with major comment 2, to which we provided answers (see above).

Minor points:
- Figs 1,3,4 are more suitable for supplement.
Response: We understand the reviewer. We have moved Figs 3 and 4 to now Supplementary Figures S2 and S3. However, we believe that Figure 1 highlights the genomic sequences of WC11 that are deleted or diverge from strain C500. We believe that this figure should be kept in the main manuscript.

- For intravenous injection, infected tissue culture cells are used, while the authors refer to pfu for intranasal infection?
Response:
Indeed, we used infected live cells for intravenous infection (revised Figs 6A-C , 7 and 8). We chose to inject the same quantity of cells showing an CPE >90% and provide now the results of infectious centers (ICs) generated for each viral inoculum (lines 565-570, revised manuscript). Although ICs numbers were about 5 times higher in WC11 and A7^{STOP} compared to WT and A8^{STOP} viruses, only cells infected with the WT virus could induce MCF.
For intranasal infections, we used concentrated cell-free virus and PFU were measured by plaque assay before inoculation in 500 uL in each nostril of the rabbits.
